# The Aching Tooth

**Published:** 1839-12

**Authors:** Giles McQuiggin


### THE ACHING TOOTH,

[ BY GILES McQUIGGIN. ]

Why art thou so rebellious, raging tooth?
Why break the peace that pleasantly prevailed,
And raise such warfare in thy warm abode?
There's room enough for thee, and chance as fair
As any of thy fellows may possess;
And there you all might dwell, a happy band,
In the firm brotherhood that Nature formed,
When she permitted you to take the place
Of that confederacy which she had tried,
And proved too weak to stand the powerful test
Of spreading bone and sinew:—on the soil
Where reigned and ruled the Aborigines
That were your predecessors, you have pow'r
To cluster unmolested; succeeding foes
May never push you from your gifted rights.

You form a compact,—leagued for life and death;
There you should stand in undivided strength,
And hail the friendly visitant that comes
To nourish and sustain you; and repel
With greater energy, the foreign foe
That dares intrude on your domestic peace.

What cause have you for quarrel in your ranks?
What party feuds to rouse you up for fight?
Or why should Cuspidatus ask a right
That to Incisor he'd refuse to grant?
Or why Incisor, seek a place of pow'r
To which his friend Molar might not aspire?
'Tis vain to talk of preference or place,
In a society so firmly bound,

And in such close connexion ;—one and all,
Should spurn the nullifier's hated cause,
And stick to *union* to the very last.

And what are names or offices to you ?
Or what care you for principle or laws,
Save those which Nature in her wisdom gave
To regulate her household ;—they belong
As well to you, as to unnumbered throngs,
That pass through being's changes to decay.

The badge of honor that the Whig may wear,
Or title that the Democrat may boast,
Hath no high preference for you to claim ;
You have no principle for which to fight,
As ever changing politicians have,
Who madly sacrifice a thousand times
In saving once, from foul pollution's touch,
The principle they quarrel to sustain.

Your only dread need be the heartless quack,
That like a greedy Vampire, seeks to draw
Blood from your mangled socket, and to thrust
His plundering talons in your owner's purse.
Or it may be, perchance, that he may drive
A plug of composition through your crown,
Or closely pack his tin, deep in your heart ;
And for the wretched deed, demand his price,
And nothing less than gold will suit his taste.

Now seeing these are truths, that I have sung,
A few of multitudes that might be told,
Why may you not all live in peace and love,
And help each other in your daily toil ?
You'd better do it ;—and you aching one,
I warn you once again ;—your raging stop,
Or by your hopes of custard, I declare,
That I shall turn against you, and raise up
With all my might, the cry, *expunge ! expunge ! !*

